# Phenotypical and functional characterization of alveolar macrophage subpopulations in the lungs of NO_2_-exposed rats

**DOI:** 10.1186/1465-9921-7-4

**Published:** 2006-01-06

**Authors:** Holger Garn, Anette Siese, Sabine Stumpf, Anka Wensing, Harald Renz, Diethard Gemsa

**Affiliations:** 1 Department of Clinical Chemistry and Molecular Diagnostics, Philipps University of Marburg, Biomedical Research Center, Hans-Meerwein-Str., 35043 Marburg, Germany; 2 Institute of Immunology, Philipps University of Marburg, Robert-Koch-Str. 17, 35037 Marburg, Germany

## Abstract

**Background:**

Alveolar macrophages (AM) are known to play an important role in the regulation of inflammatory reactions in the lung, e.g. during the development of chronic lung diseases. Exposure of rats to NO_2 _has recently been shown to induce a shift in the activation type of AM that is characterized by reduced TNF-α and increased IL-10 production. So far it is unclear, whether a functional shift in the already present AM population or the occurrence of a new, phenotypically different AM population is responsible for these observations.

**Methods:**

AM from rat and mice were analyzed by flow cytometry for surface marker expression and in vivo staining with PKH26 was applied to characterize newly recruited macrophages. Following magnetic bead separation, AM subpopulations were further analyzed for cytokine, inducible NO synthase (iNOS) and matrix metalloproteinase (MMP) mRNA expression using quantitative RT-PCR. Following in vitro stimulation, cytokines were quantitated in the culture supernatants by ELISA.

**Results:**

In untreated rats the majority of AM showed a low expression of the surface antigen ED7 (CD11b) and a high ED9 (CD172) expression (ED7^-^/ED9^high^). In contrast, NO_2 _exposure induced the occurrence of a subpopulation characterized by the marker combination ED7^+^/ED9^low^. Comparable changes were observed in mice and by in vivo labeling of resident AM using the dye PKH26 we could demonstrate that CD11b positive cells mainly comprise newly recruited AM. Subsequent functional analyses of separated AM subpopulations of the rat revealed that ED7^+ ^cells showed an increased expression and production of the antiinflammatory cytokine IL-10 whereas TNF-α production was lower compared to ED7^- ^AM. However, iNOS and IL-12 expression were also increased in the ED7^+ ^subpopulation. In addition, these cells showed a significantly higher mRNA expression for the matrix metalloproteinases MMP-7, -8, -9, and -12.

**Conclusion:**

NO_2 _exposure induces the infiltration of an AM subpopulation that, on the one hand may exert antiinflammatory functions by the production of high amounts of IL-10 but on the other hand may contribute to the pathology of NO_2_-induced lung damage by selective expression of certain matrix metalloproteinases.

## Background

The special situation in the lung, that exposes an epithelial surface of about 200 m^2 ^to the environment, requires effective defense mechanisms to safe the organism from the entry of foreign substances including pathogenic microorganisms. Indeed, the mammalian lung is equipped with a variety of defense systems that include mechanical and chemical barriers (e.g. cough reflex, mucociliary escalator, mucus, surfactant, lysozyme, defensins) as well as mechanisms of the innate and adaptive immunity (e.g. macrophages, dendritic cells, secretory IgA, bronchus-associated lymphatic tissue) [1,2]. Invading foreign materials may pass into different parts of the airways or even the lung parenchyma due to different physical and chemical properties. Therfore, certain components of the pulmonary defense system are localized at different quantities in the several parts of the lung and within the distal airways and the lung parenchyma macrophages comprise the most important cellular structures of this system [3].

Even though macrophages may occur in different localizations in the lung, alveolar macrophages (AM) are the best characterized pulmonary macrophage population [4,5]. Their special localization outside the epithelial barrier requires a specific adaptation to this environment and, indeed, AM differ in certain phenotypical and functional parameters not only from macrophages from other organs [6,7] but also from interstitial pulmonary macrophages [4,8]. On the one hand they are characterized by a higher capacity to phagocytose foreign material, increased production of reactive oxygen and nitrogen species and of the pleiotropic cytokine TNF-α. In contrast, they release reduced amounts of the proinflammatory cytokines IL-1β and IL-6 and show only a weak surface expression of MHC-class-II molecules and costimulatory molecules such as CD80 and CD86 [9]. These properties imply, that AM are very effective in the defense of microbial invaders, however, do not necessarily induce an inflammatory reaction or initiate an adaptive immune response [10]. With this respect, AM fulfill rather "classical" macrophage functions, i.e. direct defense of microorganisms and show only poor immunostimulating properties. In fact, they may even induce reversible anergy in T lymphocytes [11].

The situation may change significantly when an inflammatory reaction is induced. For example, AM with a rather monocytic phenotype appear following intratracheal administration of LPS or the CXC chemokine MCP-1 [12]. Using a rat NO_2 _exposure model, we recently demonstrated a reduced capacity of AM from exposed animals to produce superoxide radicals following in vitro stimulation with zymosan as phagocytic stimulus [13]. Moreover, AM from these animals showed a shift to an alternatively activated phenotype, mainly characterized by a reduced expression of the proinflammatory cytokines TNF-α and IL-1β and a significantly increased expression and production of the antiinflammatory cytokine IL-10 [14]. So far, it is not clear whether these changes are due to the appearance of a phenotypical different AM subpopulation or due to a functional shift in the already present AM population.

Therefore, the aim of the present study was to investigate whether phenotypically different AM subpopulations are present in the lung following NO_2 _exposure and whether these AM subpopulations show distinct functional properties. In fact we are able to show, that a phenotypically different AM subpopulation occurs in the lungs of NO_2_-exposed animals due to new infiltration. These cells show functional differences to already present AM with respect to mediator mRNA expression and production as well as mRNA expression for several matrix metalloproteinases.

## Materials and methods

### Animal exposure

Fischer344 rats were obtained from Charles River Wiga (Sulzfeld, Germany) at a body weight of about 120 g and C57BL/6 mice were purchased through Harlan Winkelmann (Borchen, Germany) at an age of 6 – 8 weeks. The animals were housed in wire cages at room temperatures in a 12-12 hours light-dark cycle and given food and water ad libitum.

Groups of rats were continuously exposed to 10 ppm NO_2 _for 24 h, 3 and 20 days, control animals breathed normal air. Exposure regimes were designed that animals of all exposure groups could be analyzed simultaneously. Mice were exposed for 7 days. Exposure was carried out in air-tight chambers having a total volume of 60 l and equipped with in- and outlet for the gas mixture and a ventilator to ensure equal distribution of the gas atmosphere throughout the whole chamber. NO_2 _(Messer-Griesheim, Duisburg, Germany) was adjusted to a final concentration of 10 ppm by mixing with compressed air and directed through the chambers at a constant gas flow of 15 l/min. NO_2 _concentration was controlled at least twice a day using a NO_2_-sensitive electrochemical element (ECS 102-1, MPSensor Systems, Munich, Germany). Exposures were performed at temperatures of 22 ± 2°C and a relative humidity of 50 ± 5 %. Animal housing conditions and NO_2 _exposure met German and International Guidelines.

### Bronchoalveolar lavage

Following anesthetization by intraperitoneal application of sodium pentobarbital (100 mg/kg body weight; Narcoren^®^, Merial GmbH, Hallbergmoos, Germany) mixed with 100 IU heparin (Liquemin^®^N, Roche, Mannheim, Germany) the tracheas were cannulated and the animals were thoracotomized. The lungs were perfused via the pulmonary artery with prewarmed (37°C) perfusion buffer (PBS + Ca^2+^, Mg^2+ ^supplemented with 10 mM HEPES, 50 μg/ml gentamicin and 10 U/ml penicillin, pH 7.4) until they became white and hearts and lungs were removed en bloc. Finally, lungs were lavaged extracorporally 6 times with 8 ml lavage buffer (Ca^2+^/Mg^2+^-free PBS with 10 mM Hepes, 0.2 mM EGTA, 50 μg/ml gentamicin and 10 U/ml penicillin, pH 7.4) which was allowed to passively run out after each instillation while gentle massaging the lung. Bronchoalveolar lavage fluid was centrifuged at 300 × g for 10 min at 4°C to obtain alveolar cells. Contaminating red blood cells were eliminated by hypotonic lysis for 30 seconds with double-distilled water. Remaining cells were washed twice in PBS.

### FACS analysis

Surface marker expression of AM was investigated by labeling of the cells with several primary antibodies directed to rat myeloid cell epitopes (kindly provided by Dr. Steiniger, Institute of Anatomy, Philipps University of Marburg; see Table 2) combined with a signal amplification system to overcome draw-backs evoked by the high AM autofluorescence and subsequent flow cytometric analysis. Briefly, cells were suspended in FACS buffer (PBS supplemented with 1% fetal calf serum and 0.1% sodium azide) at a concentration of 2 × 10^6 ^cells/ml and 250 μl of the cell suspensions were labeled with 50 μl of the appropriately diluted, unlabeled primary antibody. Bound antibodies were than detected by addition of a biotinylated goat anti-mouse antibody (Becton Dickinson – Pharmingen, Heidelberg, Germany) followed by phycoerythrin (PE)-conjugated streptavidin (Becton Dickinson – Pharmingen). This complex was then incubated with a biotinylated anti-streptavidin antibody (Vector, Burlingame, CA) and, finally, all free biotin binding sites were labeled by repeated addition of PE-labeled streptavidin.

Mouse AM were labeled with anti-mouse CD11b-biotin and fluorescein isothiocyanate (FITC)-labeled strepatvidin as secondary reagent (both purchased from Becton Dickinson – Pharmingen) and the macrophage-specific antibody F4/80 conjugated to Alexa647 (Caltag, Hamburg, Germany).

All incubations were performed at 4°C for 30 min and after each incubation, unbound reagents were washed out by three washing steps with FACS buffer. Stained cells were finally suspended in 250 μl FACS fixation buffer (FACS buffer plus 1% formaldehyde) and 250 μl of azide free Diluid^® ^(J.T. Baker B.V., Deventer, The Netherlands) were added prior to FACS analysis. Appropriate controls were performed to ensure the specificity of the labeling reactions including use of irrelevant isotype control immunoglobulins and omission of key reagents.

Flow cytometric analysis of stained cells was carried out using a FACScan (Becton Dickinson). A forward scatter life gate was set and 5,000 events were measured for each sample using FACScan Plus software. Data analysis was performed with the PC-compatible FlowMate software (Dako A/S, Glostrup, Denmark).

### Preparation of purified AM subpopulations by magnetic bead separation

AM subpopulations were separated by a two-step purification protocol using the MACS magnetic cell sorting system (Miltenyi Biotec, Bergisch Gladbach, Germany). In the first step, neutrophils and T cells were removed to obtain purified total AM that were further separated in a second step in ED7^- ^and ED7^+ ^AM. Therefore, BAL cells were resuspended in 5 ml MACS buffer (PBS without Ca^2+^/Mg^2+ ^+ 2 mM EDTA + 0.5% bovine serum albumin) and subsequently filtered through 75 μm and 30 μm filters to remove cell clumps. After washing and resuspension in 5 ml MACS buffer, 10 μl of HIS-48-biotin (labels rat neutrophil granulocytes; Becton Dickinson – Pharmingen) antibody solution were added. Cell suspensions were incubated at 4°C on a roller shaker for 20 min and washed twice in MACS buffer. Subsequently, cells were suspended in 80 μl MACS buffer plus 10 μl streptavidin-beads and 10 μl rat pan T cell beads. After another 20 min of incubation, cells were washed, suspended in 0.5 ml MACS buffer and applied to MACS-MS columns that were placed in an OctoMACS separation unit (all materials from Miltenyi). Subsequently, the columns were washed three times with 0.5 ml MACS buffer. Cells in the pooled flow throughs represented purified total AM with a purity of >99 %. Similar to the first step protocol, these cells were than labeled with the ED-7 antibody (Serotec, Duesseldorf, Germany) followed by anti-mouse-IgG beads (Miltenyi) and separated on MACS-MS columns. Cells in the flow throughs were collected as ED7^- ^AM, and ED7^+ ^AM were obtained by washing the columns after removal from the magnet. Finally, cells were washed and resuspended in the respective buffer or medium for subsequent applications.

### In vivo labeling of resident AM with PKH26

Three days prior to the initiation of NO_2_- or sham-exposure, 100 μl of a 300 μM solution of PKH26 dissolved in Diluent C (PKH26 Red Fluorescent Phagocytic Cell Linker Kit, Sigma, Deisenhofen, Germany) were intravenously injected into mice, resulting in an estimated serum concentration of 15 μM according to Maus et al. [12].

### Quantitative reverse transcriptase polymerase chain reaction

Total RNA from purified AMs was prepared using the RNeasy Total RNA Mini Kit (Qiagen, Hilden, Germany) according to manufacturer's protocol. For first-strand cDNA synthesis, RNA was treated with DNase I (Gibco – Invitrogen, Groningen, The Netherlands) and subsequently reverse-transcribed using an oligo(dT)_20 _primer (MWG Biotech, Ebersberg, Germany) and Omniscript Reverse Transcriptase (Qiagen). All procedures were carried out according to supplier's recommendations.

Primer sequences were generated from the respective mRNA sequences obtained from the European Molecular Biology Laboratory (EMBL) gene bank and primers were synthesized by MWG Biotech. Primer sequences are summarized in Table 1. Quantitative LightCycler PCR was performed by use of the QuantiTect^® ^SYBR^® ^Green PCR Kit (Qiagen). Therefore, 12.5 μl QuantiTect^® ^SYBR^® ^Green Master Mix, 0.5 μl of each primer at a concentration of 50 pmol/μl and 10.5 μl water were added to 1 μl of cDNA, standard or water (negative control). 20 μl of each mix were transferred into LightCycler capillaries (Roche, Mannheim, Germany) that were subjected to the following temperature profile within the LightCycler equipment (Roche): initial 15 min at 95°C to activate the enzyme, and 55 cycles of 95°C (15 sec) – 60°C (30 sec) – 72°C (15 sec). Finally, product identity was verified by melting curve analysis. Calculation of crossing points was performed using the second derivative maximum method (included in LightCycler software) for the unknown samples and for DNA standards of known concentrations generated from purified PCR-products of the respective gene. Unknown sample concentrations were than calculated from the standard curve. Sample equality was confirmed by comparable expression of the housekeeping gene L32.

**Table 1:** Primer sequences.

Gene	Primer	Sequence
TNF-α	sense	5'- TCC CAA ATG GGC TCC CTC TC -3'
	antisense	5'- AAA TGG CAA ACC GGC TGA CG -3'
IL-10	sense	5'- CCA TGG CCC AGA AAT CAA GG -3'
	antisense	5'- TCT TCA CCT GCT CCA CTG CC -3'
iNOS	sense	5'- TTG CCA CGG AAG AGA CGC AC -3'
	antisense	5'- CAG GCA CAC GCA ATG ATG GG -3'
IL-12 p40	sense	5'- GTT CTT CGT CCG CAT CCA GC -3'
	antisense	5'- GCA TTG GAC TTC GGC AGA GG -3'
MMP-2	sense	5'- AGT TCC CGT TCC GCT TCC AG -3'
	antisense	5'- CCA CAC CTT GCC ATC GCT TC -3'
MMP-7	sense	5'- TGC CGG AGA CTG GAA AGC TG -3'
	antisense	5'- GGT GCA AAG GCA TGG CCT AG -3'
MMP-8	sense	5'- TGC CCG ACT CTG GTG ATT TC -3'
	antisense	5'- GGG TTG ATG GCA CAC TCC AG -3'
MMP-9	sense	5'- ACT TGC CGC GAG ACG TGA TC -3'
	antisense	5'- TTG CCG TCG AAG GGA TAC CC -3'
MMP-12	sense	5'- TCG ATG TGG AGT GCC TGA TG -3'
	antisense	5'- ATC CGC ACG CTT CAT GTC TG -3'
L32	sense	5'- AAG CGA AAC TGG CGG AAA CC -3'
	antisense	5'- CTG GCG TTG GGA TTG GTG AC -3'

### In vitro stimulation of BAL cells

Separated ED7^- ^and ED7^+ ^AM were washed twice in Ca^2+^/Mg^2+^-free PBS and were suspended in RPMI 1640 (Linaris, Bettingen, Germany) supplemented with 2 mM L-glutamine, 10 mM HEPES, 1 mM sodium pyruvate, 1 × non-essential amino acids, 100 U/ml penicillin and 100 μg/ml streptomycin (all purchased from Life Technologies, Gaithersburg, MD) and 1 % fetal calf serum (FCS, Biochrom, Berlin, Germany). The number of living cells was determined using the CASY^®^1 Cell Counting System (Schärfe Systems, Reutlingen, Germany) and AMs were incubated at a final concentration of 1 × 10^6 ^cells/ml in 48-well cell culture plates (Costar, Corning, NY) at a total volume of 250 μl. Cell cultures were performed in the absence or presence of LPS from E. coli O127:B8 (Difco Laboratories, Chicago, MI) at 37°C in a humid atmosphere containing 5% CO_2_. Cells were allowed to adhere to the culture plate surface for about 1 hour before LPS (100 ng/ml) was added. Cell culture supernatants were collected after 24 hours of culture and stored until use for mediator quantitation at -20°C.

### Cytokine quantitation in cell culture supernatants

Cell culture supernatant TNF-α and IL-10 were measured with rat specific enzyme-linked immunosorbent assays (ELISAs) using matched antibody pairs with monoclonal capture and biotinylated detection antibodies and recombinant cytokines (all purchased from Becton Dickinson – Pharmingen) as standards. ELISAs were performed according to a recently described protocol [15] using peroxidase-labeled streptavidin (Roche, Heidelberg, Germany) and o-phenylendiamine (Sigma, Deisenhofen, Germany) as substrate.

IL-12 p70 was quantitated using a commercially available ELISA to rat IL-12 p70 obtained from Biosource (Nivelles, Belgium) that was carried out according to the instructions of the manufacturer.

## Results

### Phenotypical characterization of AM of NO_2_-exposed rats

First we analyzed by flow cytometry the expression of several surface molecules on AM obtained from rats exposed to NO_2 _for different times. Since AM are known to exert a high degree of autofluorescence that often interferes with the detection of surface molecules by FACS analysis we developed an amplifying system to improve the signal to background (autofluorescence) ratio. For this method, cells were initially labeled with the respective unconjugated primary antibody (all generated in the mouse) that was then detected by a biotinylated secondary antibody (goat anti-mouse IgG) followed by streptavidin-PE. This complex was now incubated with an anti-streptavidin antibody also labeled with biotin and, finally, streptavidin-PE was added again to cover all free biotin binding sites. The application of this method enabled us to demonstrate the expression of surface molecules on alveolar macrophages that were not to be detected with conventional staining methods.

**Figure 1:**
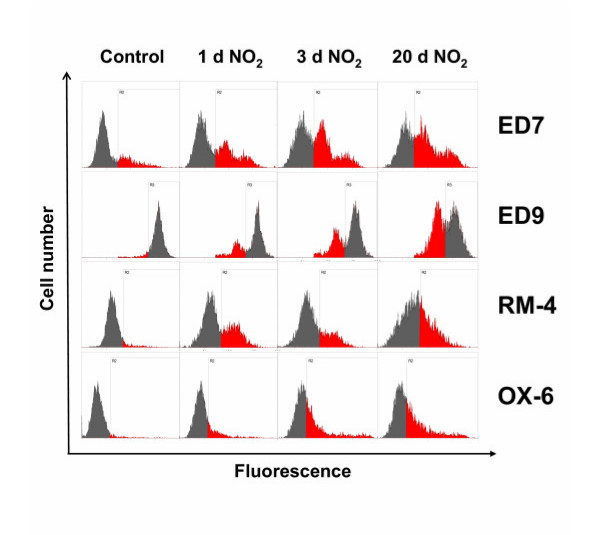
Flow cytometric analysis of AM from NO_2_-exposed and control rats. Rats were exposed to NO_2 _for the indicated times and BAL cells were stained with antibodies to ED7, ED9, RM-4, and OX-6. To overcome autofluorescence signals, primary antibodies were detected using a biotin-PE/streptavidin-anti-streptavidin enhancing system and labeling of AM was analyzed by flow cytometry following gating by help of forward and sideward scatter properties. Shown are representative results of at least six animals per group.

Having this method available we characterized normal AM of the rat using a number of antibodies that have been described or assumed to react with cells of the myeloid hematopoetic lineage and could demonstrate the surface expression of different molecules on AM as summarized in Table 2. In addition, for certain markers we were able to detect differences in the expression level in AM obtained from NO_2_-exposed rats in comparison to those obtained from untreated control animals (see Table 2 and Figure 1). With exception of ED9, AM from NO_2_-exposed animals showed always a higher expression of the respective surface marker when compared to cells from controls. The most remarkable differences were demonstrated using the antibodies ED7, ED9, RM-4 and OX6. Staining with ED7 clearly revealed the increasing occurrence of a second AM subpopulation that was characterized by a higher ED7 antigen expression, perhaps themselves representing two populations with medium and high ED7 expression. In contrast, ED9 showed a strong staining of all AM from treated and untreated animals, however, a subpopulation showing a slightly lower ED9 expression was found the longer the animals had been exposed to NO_2_. An increased surface expression was also found for the marker RM-4 and for MHC-class-II molecules, as detected by the antibody OX-6, in AM from exposed rats (Figure 1).

**Table 2:** Overview of cell surface expression of several cell surface molecules on rat alveolar macrophages and detection of differential expression in AM from NO_2_-exposed rats in comparison to AM from untreated controls. Expression analysis was performed by flow cytometry following staining of cells with the respective primary antibody and a signal amplification system.

**Antibody**	**Antigen/Cell population**	**Expression**	**Differences**
**1A29**	ICAM-1 (CD54)	medium	medium
**1C7**	mononuclear phagocytes (CD68 ?)	medium	medium
**3.2.3.**	NKR-P1 (CD161)	weak	no
**3A12**	PECAM-1 (CD31)	weak	no
**5F10**	VCAM-1	no	
**ART18**	IL-2 receptor	no	
**ART65**	IL-2 receptor	no	
**ED2**	macrophage subset (no monocytes)	no	
**ED3**	macrophage subset (no monocytes)	no	
**ED4**	macrophages	medium	no
**ED7**	macrophage subset (CD11/CD18; CR3)	medium	strong
**ED8**	macrophage subset (CD11/CD18; CR3)	medium	small
**ED9**	macrophage subset (SIRP_α_, CD172a)	strong	medium
**KIM2R**	mature tissue macrophages	no	
**MAR3**	macrophage subset	no	
**Ox2**	CD200	no	
**Ox26**	transferrin receptor (CD71)	no	
**Ox3**	MHC-II (I-A like)	weak	small
**Ox4**	MHC-II (I-A like)	weak	small
**Ox41**	macrophages, DCs, PMNs (SIRP)	no	
**Ox50**	hyaluronic acid receptor (CD44)	medium	small
**Ox52**	activated monocytes		
**Ox6**	MHC-II (RT1.B; I-A)	weak	medium
**Ox62**	DC subpopulation	no	
**Ox8**	CD8_α_	no	
**Ox85**	L-selectin (CD62L)	no	
**RM-1**	monocytes/macrophages/DCs/PMNs	strong	small
**RM-4**	all macrophages (no monocytes)	medium	strong
**RMA**	macrophage subset (120 kDa antigen)	medium	medium
**RP-1**	neutrophiles (intracellular)	no	
**RP-3**	neutrophiles (intracellular)	no	
**W3/13**	leukosialin (CD43)	no	
**WT/1**	LFA-1 (CD11a)	weak	no

**Figure 2:**
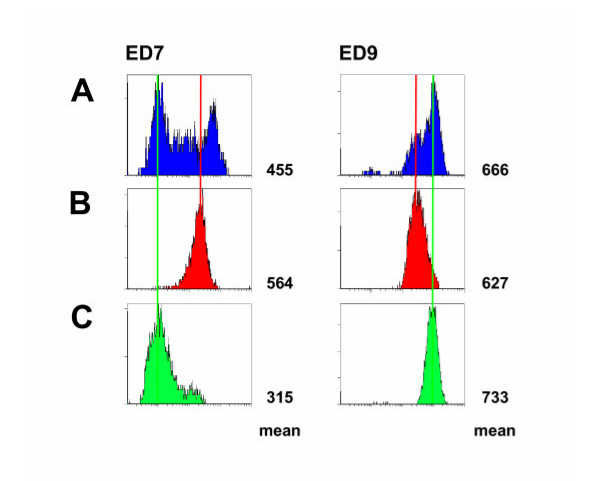
Flow cytometric analysis of ED7 and ED9 expression of AM following magnetic bead separation. AM of 3 days NO_2_-exposed rats were separated due to their expression of the cell surface molecule ED7 using magnetic bead separation. Susbsequently, ED7 (left) and ED9 (right) expression was analyzed in unseparated AM (A), ED7-positive AM (B), and ED7-negative AM (C). Numbers right of each histogram represent the mean fluorescence of the respective cell population. The figure clearly demonstrates that ED7-positive AM show a lower ED9 expression compared to ED7-negative AM. Shown is a representative data set of more than twenty animals.

The major disadvantage of the applied signal amplification method is that double staining of cells is not possible. To further characterize the observed AM subpopulations we, therefore, separated AM obtained from 3 days exposed animals that show a low expression of ED7 (further referred as ED7^-^) from those showing a high ED7 expression (ED7^+^) by use of magnetic bead separation after removing contaminating neutrophils and lymphocytes. As shown in the left panel of Figure 2 we obtained very pure AM subpopulations. These cells were now stained with the ED9 antibody combined with the described amplification system. Interestingly, we found that those AM showing a high level of ED7 expression are characterized by a reduced ED9 expression whereas the ED7^- ^AM show the higher ED9 surface expression (Figure 2, right panel). Thus, two AM subpoplations were demonstrated in the lungs of NO_2_-exposed rats that are characterized by the marker combinations ED7^+^/ED9^low ^and ED7^-^/ED^high^, in the following still referred as ED7^+ ^and ED7^- ^AM, respectively.

### Origin of AM subpopulations in NO_2_-exposed animals

**Figure 3:**
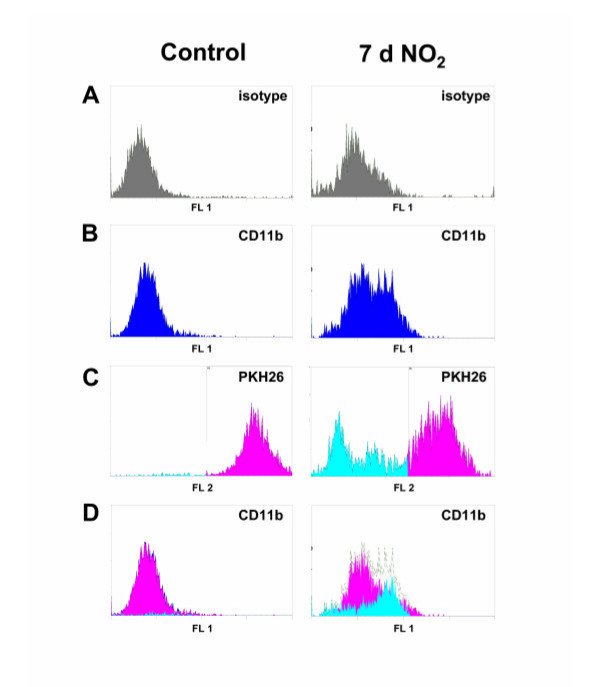
FACS analysis of CD11b and PKH26 labeling of AM from NO_2_-expsoed C57BL/6 mice. Mice were intravenously given PKH26 in combination with diluent C three days prior to onset of a seven days NO_2_-exposure. Afterwards, AM were stained with F4/80-Alexa647 and CD11b-FITC. Isotype control (A), CD11b (B) and PKH26 (C) staining was subsequently analyzed by flow cytometry within the F4/80-positive cell population. (C) The proportion of PKH26-negative cells is shown in blue. Part (D) shows a separate analysis of CD11b-expression in PKH26-negative (blue histogram) and PKH26-positive AM (pink histogram) thereby clearly demonstrating that the CD11b-positive cell population mainly consists of PKH26-negative, newly recruited AM. Shown are representative results of eight animals per group.

The occurrence of phenotypically different AM subpopulations may either be explained by a functional shift of already present AM or by the infiltration of macrophages that already represent the different phenotype. To address this question we applied the recently described method of in vivo labeling of resident AM by use of the fluorescent cell tracer PKH26 [12]. When intravenously applied in combination with a specific diluent, this dye is able to label phagocytic cells within the organs, e.g. AM of the lungs, without a significant staining of blood monocytes. However, since this model is only applicable for the mouse, we switched to the mouse model for these investigations. Since we have recently shown that mice show a slower development of the inflammatory reaction towards NO_2 _[16], mice were exposed for 7 days for these analyses. Following this treatment, also in mice an AM subpopulation was observed that revealed an increased expression level of CD11b, the mouse homologue to ED7 (Fig. 3B). To analyze the origin of these cells, mice were treated with PKH26 three days prior to the onset of the NO_2_- or sham-exposure. At this time point, almost 100 % of the AM were positively stained whereas blood monocytes appeared PKH26-negative (data not shown). Whereas this situation did not change in the sham-exposed control group, a significant portion of PKH26-negative, newly recruited AM were observed in the lungs of NO_2_-exposed mice (Fig. 3C). A separate analysis of PKH26-positive and PKH26-negative cells revealed that the latter population was indeed characterized by a higher expression of the surface marker CD11b indicating that the CD11b-positive AM subpopulation mainly originated from newly recruited macrophages (Figure 3D).

### Cytokine and iNOS mRNA expression in separated AM subpopulations

**Figure 4:**
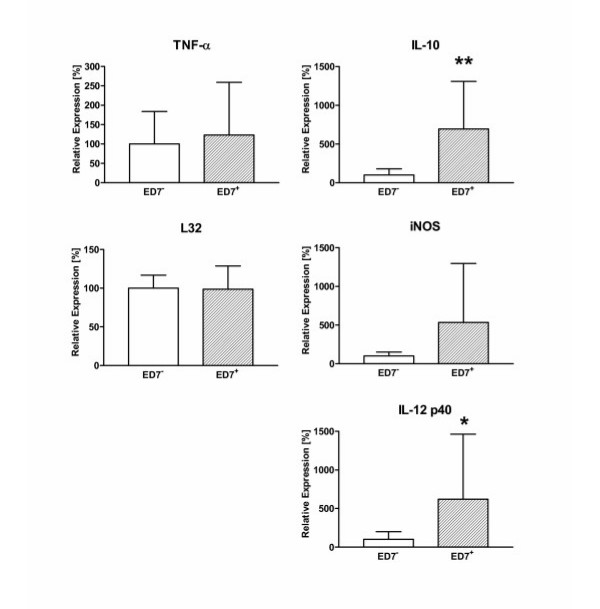
Cytokine and iNOS mRNA expression in AM subpopulations of NO_2_-exposed rats. ED7-positive and ED7-negative AM were separated from 3 days NO_2_-exposed rats and total RNA was prepared immediately after cell separation. Cytokine (TNF-α, IL-10, IL-12 p40) and iNOS mRNA expression was analyzed by quantitative RT-PCR with L32 as house-keeping gene control in ED7-negative (blank bars) and ED7-positive AM (hatched bars). Data are presented as relative expression with mean expression in ED7-negative AM was 100 %. Shown are mean ± SD of six animals per group. Significance of differences was tested using the U-test according to Mann and Whitney and is indicated by * for p < 0.05 or ** for p < 0.01.

For functional analysis of the two phenotypically different AM subpopulations we first compared the mRNA expression for several macrophage-derived mediators that are involved in the regulation of inflammatory responses. Therefore, ED7^+ ^and ED7^- ^AM of the rat were separated from the lungs of 3 days exposed animals. Total RNA was immediately isolated and following cDNA synthesis mediator mRNA expression was analyzed by quantitative PCR. As shown in Figure 4, no differences were observed between the two AM subpopulations in the expression of the proinflammatory cytokine TNF-α. However, significantly increased mRNA levels were found in the ED7^+ ^population for IL-12 p40 and iNOS. Interestingly, the expression of the antiinflammatory cytokine IL-10 was also higher in the ED7^+ ^AM subpopulation (Fig. 4).

### Cytokine release by AM subpopulations following in vitro stimulation

**Figure 5:**
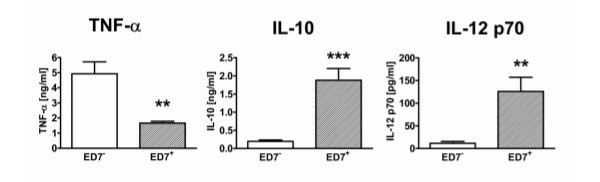
Differential cytokine production by LPS-stimulated AM subpopulations of NO_2_-exposed rats. ED7-positive and ED7-negative AM were separated from 3 days NO_2_-exposed rats and cultured in vitro for 24 hours in the presence of 100 μg LPS. Subsequently, TNF-α, IL-10, and IL-12 p70 were quantitated in the culture supernatants of ED7-negative (blank bars) and ED7-positive AM (hatched bars) by ELISA. Data are presented as mean ± SD of at least six animals per group. Significance of differences was tested using Students t-test and is indicated by ** for p < 0.01 or *** for p < 0.001.

To confirm the importance of the gene expression data we stimulated separated AM in vitro with 100 ng/ml LPS and analyzed the release of cytokines in the 24 h culture supernatants. When investigating proinflammatory cytokines we found that TNF-α was released at significantly higher amounts by AM of the ED7^- ^subpopulation whereas IL-12 p70 was released at higher levels by the ED7^+ ^subpopulation. However, the most important difference was observed for IL-10 that was detected in more than 100-fold amounts in the supernatants of ED7^+ ^AM when compared to the ED7^- ^subpopulation (Fig. 5).

### MMP mRNA expression in separated AM subpopulations

**Figure 6:**
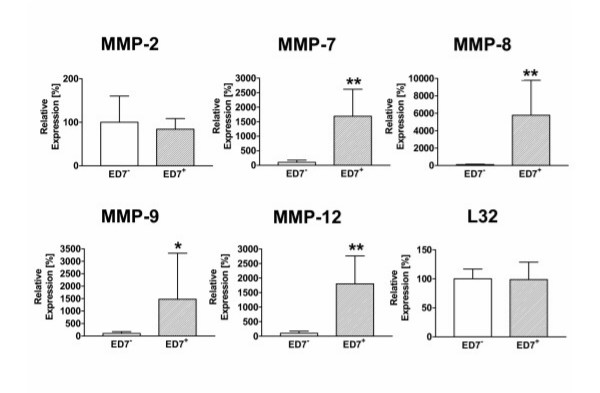
mRNA expression for several MMPs in AM subpopulations of NO_2_-exposed rats. ED7-positive and ED7-negative AM were separated from 3 days NO_2_-exposed rats and total RNA was prepared immediately after cell separation. MMP-2, -7, -8, -9, and -12 mRNA expression was analyzed by quantitative RT-PCR with L32 as house-keeping gene control in ED7-negative (blankbars) and ED7-positive AM (hatched bars). Data are presented as relative expression with mean expression in ED7-negative AM was 100 %. Shown are mean ± SD of six animals per group. Significance of differences was tested using the U-test according to Mann and Whitney and is indicated by * for p < 0.05 or ** for p < 0.01.

In the context of an oxidant-induced inflammatory reaction in the lung AM are not only involved in the regulation of the inflammatory reaction by release of respective mediators but may also produce factors such as MMPs that may contribute to tissue remodelling and also lung damage under these conditions. We therefore investigated whether a specific subpopulation of AM is responsible for the expression of several metalloproteinases. The results of these analyses are summarized in Figure 6. With exception of MMP-2, that showed a comparable expression in both AM subpopulations, mRNA for all other tested MMPs (MMP-7, MMP-8, MMP-9, and MMP-12) were almost not detectable in the ED7^- ^subpopulation but were found at significantly elevated levels in the ED7^+ ^AM subpopulation.

## Discussion

Exposure of rodents to NO_2 _have been shown to induce inflammatory reactions in the lung that have several features in common with the situation observed in patients that suffer from inflammatory lung diseases such as chronic obstructive lung disease (COPD). Due to it's poor water solubility NO_2 _may reach distal parts of the lung including small airways and lung parenchyma where it causes histopathological and functional changes. These alterations comprise histomorphological changes in lung parenchyma and vasculature [17,18] with increased vascular permeability [14], loss of cilia in the airway epithelium [19], hypertrophy of bronchial epithelial cells [20], and mucus hypersecretion due to a hyperplasia of goblet cells. In addition, several changes in surfactant metabolism were described [21,22] and a replacement of type-I-pneumocytes by type-II-cells was observed [20]. Moreover, prolonged exposure to NO_2 _may also cause changes in lung function such as limitation of airflow and increased expiration time that are indicative for the occurrence of airway obstruction [23] and may finally even lead to the development of emphysema [24,25]. Especially the last features are major characteristics of human COPD. As also observed in these patients, macrophages and neutrophil granulocytes are the most important inflammatory cell populations [25,26]. Using the identical NO_2 _exposure model as applied for the investigations described here we could recently demonstrate that neutrophils show an immediate infiltration and their number peaks in the BAL already at three days after onset of the exposure in rats [14]. Even though mice show a slower development of inflammatory changes [16], macrophages play the dominant role over the whole observation period in both species. With exception of day one in rats, significantly increased alveolar macrophage numbers have been observed over the whole observation period in rat and mice, thereby representing the major cell population at all time points [14]. However, only little is known about the role that AM play in the pathogenesis of chronic inflammatory lung diseases especially at early stages of their development.

In the present study we could clearly demonstrate that a new phenotypically different AM subpopulation occurs in the lungs of rats and mice under the influence of oxidative/nitrosative stress exerted by exposure of the animals to NO_2_. Using PKH labeling of resident AM in mice we were able to show, that these macrophages represent newly recruited macrophages, a mechanism that is assumed to be similar in rats. These macrophages differ from already present AM by a higher expression of the surface marker ED7 (in rat) or its murine homologue CD11b. Interestingly, an increased expression of CD11b was also observed in AM from smokers [27]. In addition, other surface markers are also differentially expressed in AM from control and NO_2_-exposed animals, e.g. ED9, RM-4 and MHC-class-II molecules, at least in the rat. AM are known to normally show a low expression of CD11b even though this molecule is a typical surface marker of cells of the monocyte/macrophage lineage in the blood and other tissues [28]. Thus, the limited CD11b expression seems to be a sign of tissue specific activation of AM that also show an elevated expression of the transcription factor PU.1 [29], a differential expression of protein kinase C isoforms [30] and a decreased DNA binding capacity of the transcription factor AP-1 [31] when compared to macrophages from other tissues. In addition, the proteome of AM differs significantly from that of blood monocytes [32]. Perhaps, AM-specific differentiation signals are underrepresented during an inflammatory process in the lung or these signals may not properly influence newly infiltrating macrophages under these circumstances. As a consequence, these alterations may lead to a different phenotype of AM that enter the lung during an inflammatory process in comparison to macrophages that infiltrate under non-pathological conditions. However, very recent data also suggest the existence of two phenotypically different monocyte populations that selectively enter healthy or inflamed tissue areas [33,34]. This would imply that the described AM subpopulations originated from already different monocyte subpopulations.

In the model presented here, newly recruited AM seem to have a dual role with respect to regulatory and effector functions. A major feature of these cells is their high expression and production of IL-10 which is in contrast to resident AM that do only poorly produce this cytokine even following LPS stimulation [35]. IL-10 is known to exert antiinflammatory properties [36] and, therefore, ED7^+ ^AM seem to play a role in the control of the inflammatory reaction. On the other hand these ED7^+ ^AM also produce higher amounts of IL-12, a cytokine that is involved in the activation of T helper 1 (Th1) lymphocytes [37] that in turn may amplify a macrophage-dominated inflammatory reaction. The latter mechanism is supported by observations in CCR2 knock-out mice that lack the receptor for the CC-chemokine CCL2 (MCP-1; monocyte chemotactic protein-1). These animals show diminished inflammatory reactions due to an impaired migration of monocytes into inflammatory sites associated with decreased Th1 activities [38]. In line with these findings it has also been demonstrated that these mice exert enhanced Th2 responses [39,40]. In conclusion, our findings clearly suggest that the newly recruited ED7^+ ^AM are involved in the regulation of the ongoing inflammatory process. Whether the antiinflammatory effects of IL-10 or the proinflammatory role of IL-12 (or even additional regulatory molecules) will dominate the regulatory function of ED7^+ ^AM in our model has to be investigated in future studies.

In addition, ED7^+ ^AM are not only involved in regulatory processes but may also directly act as effector cells. With this respect the selective expression of several MMPs by these macrophages was a quite interesting finding. It is known that activated granulocytes and macrophages are major producers of these proteases [41], however, to our best knowledge this is the first description that a specific inflammatory macrophage subpopulation is almost selectively responsible for the production of certain MMPs, among them MMP-9 and MMP-12. Interestingly, lung macrophages from human smokers and COPD patients have also been reported to show an increased expression of MMP-9 [42] but macrophage subpopulations were not investigated. MMP-12 seems to play an important role in the development of emphysema at least in the mouse model since absence of this specific MMP inhibits the generation of cigarette-smoke induced emphysema in MMP-12 knock out mice [43]. More recent investigations provide evidence that both, elastase activities, such as MMP-12, and collagenolytic activities, as exerted by MMP-2 and MMP-9, in combination lead to an effective destruction of lung parenchymal tissue that finally results in the generation of emphysema [44,45]. In addition, certain MMPs may also be involved in the regulation of inflammatory processes, e.g. by activation or inactivation of inflammatory mediators [46-48]. Thus, by expression of important MMPs ED7^+ ^AM may contribute to the pathology of NO_2_-induced lung damage and are further involved in the regulation of the inflammatory process.

## Conclusion

Exposure of rodents to the oxidative/nitrosative agent NO_2 _leads to the infiltration of a new AM subpopulation that phenotypically and functionally differs from resident AM. There is no doubt that these AM by release of regulatory mediators and expression of MMPs strongly influence the mechanisms that regulate the inflammatory response to the inducing agent and are directly involved in the pathologic processes induced by NO_2_. Since NO_2 _and related molecules are major components of tobacco smoke it is likely that similar processes may occur in smokers and even patients suffering from COPD. Indeed, phenotypically and functionally different macrophages have been observed in sputum of those patients [49]. These macrophages represent a different compartment of the lung, however, their occurrence implicates that similar processes as described in our animal model may also occur in humans following oxidative/nitrosative stress. If so, these newly recruited macrophages may represent an interesting target for therapeutic approaches for the treatment of chronic inflammatory diseases of the lung.

## Competing interests

The author(s) declare that they have no competing interests.

## Authors' contributions

HG conceived of and designed the study, was involved in animal exposure and cell preparation, performed FACS analysis and drafted the manuscript.

AS was involved in animal exposure and cell preparation, carried out MACS separation of AM subpopulations and performed in vitro cell stimulation and mediator analysis.

SS was responsible for animal preparation, performed mRNA expression analyses, and helped in FACS and MACS procedures.

AW performed the PKH26 experiments and was involved in subsequent FACS analyses. In addition she helped carrying out mRNA-expression analyses.

HR helped in study design and coordination as well as in preparation of the manuscript.

DG participated in the design of the experiments, its coordination and manuscript preparation.
